# Involvement of Slit–Robo signaling in the development of the posterior commissure and concomitant swimming behavior in *Xenopus laevis*

**DOI:** 10.1186/s40851-015-0029-9

**Published:** 2015-10-05

**Authors:** Yasuhiko Tosa, Kiyohito Tsukano, Tatsuya Itoyama, Mai Fukagawa, Yukako Nii, Ryota Ishikawa, Ken-ichi T. Suzuki, Makiko Fukui, Masahumi Kawaguchi, Yasunori Murakami

**Affiliations:** Graduate School of Science and Engineering, Ehime University, 2-5 Bunkyo-cho, Matsuyama, 790-8577 Japan; Graduate School of Medicine and Pharmaceutical Sciences, University of Toyama, 2630 Sugitani, Toyama, 930-0194 Japan; Graduate School of Science, Hiroshima University, 1-3-1 Kagamiyama, Higashi-Hiroshima, Hiroshima 739-8526 Japan

## Abstract

**Introduction:**

During vertebrate development, the central nervous system (CNS) has stereotyped neuronal tracts (scaffolds) that include longitudinal and commissural axonal bundles, such as the medial longitudinal fascicle or the posterior commissure (PC). As these early tracts appear to guide later-developing neurons, they are thought to provide the basic framework of vertebrate neuronal circuitry. The proper construction of these neuronal circuits is thought to be a crucial step for eliciting coordinated behaviors, as these circuits transmit sensory information to the integrative center, which produces motor commands for the effective apparatus. However, the developmental plan underlying some commissures and the evolutionary transitions they have undergone remain to be elucidated. Little is known about the role of axon guidance molecules in the elicitation of early-hatched larval behavior as well.

**Results:**

Here, we report the developmentally regulated expression pattern of axon-guidance molecules Slit2 ligand and Robo2 receptor in *Xenopus laevis* and show that treatment of *X. laevis* larvae with a *slit2*- or *robo2*-morpholino resulted in abnormal swimming behavior. We also observed an abnormal morphology of the PC, which is part of the early axonal scaffold.

**Conclusion:**

Our present findings suggest that expression patterns of *Slit2* and *Robo2* are conserved in tetrapods, and that their signaling contributes to the construction of the PC in *Xenopus*. Given that the PC also includes several types of neurons stemming from various parts of the CNS, it may represent a candidate prerequisite neuronal tract in the construction of subsequent complex neuronal circuits that trigger coordinated behavior.

## Introduction

External stimuli received by several types of sensory receptors located on the body’s surface are transferred to the peripheral nerves. Subsequently, the nerve afferents enter the brain and send information to relay nuclei, which in turn project to higher centers in which the various sensory inputs are integrated. The motor center then outputs commands to motoneurons located in the hindbrain or in the spinal cord. Thus, the construction of a precise circuit is a crucial step in eliciting appropriate behavioral responses. If such neuronal circuits are disorganized during ontogenesis, early larvae may be unable to perform coordinated body movements. In the developing vertebrate central nervous system (CNS), early-differentiating neurons extend axons toward their target regions, forming stereotyped tracts (scaffolds) consisting of longitudinal and commissural axonal bundles [1–8]. In later development, these early tracts are thought to serve as guideposts for later-developing axons [9]. The basic framework of these tracts is highly conserved in vertebrate evolution [2, 10]. The early tracts consist of longitudinal (extending along the anteroposterior axis) and commissural (connecting to the left and right side of the brain) tracts. The former include the lateral longitudinal fascicle, tracts of the postoptic commissure (TPOC), and the supraoptic tract (SOT); the latter include the anterior, habenular (HC), and posterior (PC) commissures.

Developing vertebrate brains are typically subdivided into series of segments called neuromeres, and those located in the diencephalon are called prosomeres [11–17]. It is known that some commissural bundles are located at a specific region on the neural tube corresponding to prosomeric compartments. The HCs and PCs are formed in prosomere 2 (thalamus) and prosomere 1 (pretectum), respectively, in many vertebrate groups [14]. The highly conserved framework of these commissures implies that a strictly maintained neurodevelopmental program involved in their wiring has been inherited during vertebrate evolution. In fact, some transcription factors and axon-guidance molecules have been shown play an important role in the formation of the network of these early tracts [18–20]. Previous studies have revealed that the interaction between Slit (ligand) and Robo (receptor), which acts as a repulsive guidance signal, plays an essential role in the formation of early scaffolds, e.g., the inhibition of Slit2 or Robo2 results in an abnormal morphology of the TPOC [21–23] and SOT [24]. These molecules are also involved in the formation of commissural tracts in insects [25, 26] and vertebrates (Slit:[27, 28]; Robo:[29, 30]). In zebrafish robo3 mutant, the axons of the Mauthner neuron fail to cross the midline [31]. In mammals, the Slit/Robo interaction is involved in the formation of the corpus callosum, which is a type of commissural system that connects the cerebral hemispheres [32]. The similarity of the Slit-Robo interaction in teleosts and rodents leads to the possibility that the role of slit and robo is conserved in vertebrate evolution. To study such evolutionary conservation in the vertebrate lineage, it is important to study the function of slit and robo in amphibians, as these animals are thought to have diverged between the teleost and amniote lineages. In addition, the development of locomotion patterns in larval stages is well-described in anuran species [33–35]. For these reasons, *Xenopus laevis,* an anuran species, may be a suitable model for use in phylogenetic studies and behavioral analysis in early hatched larvae. The aim of the present study is to identify the role of Slit-Robo signaling in the formation of early tracts and/or the elicitation of swimming behavior in *Xenopus*. We also tried to identify the evolutionary transition of Slit-Robo signaling. To this end, we studied the expression pattern of Slit2 and Robo2 in *Xenopus* embryos, then perturbed their signals using morpholino antisense oligonucleotides (MO) and analyzed the swimming behavior of early larvae. We found that expression domains of Slit2 and Robo2 in *Xenopus* are similar to those of amniotes, indicating that the axon guidance mechanism that depends on Slit-Robo signaling is evolutionarily conserved in the forebrain of tetrapods. We also found a disorganized swimming behavior and an abnormal morphology of the PC in both *slit2*- and *robo2*-MO-injected larvae. These results indicate that interaction between Slit2 and Robo2 is involved in the construction of the PC and the formation of neuronal element(s) that control coordinated body movement in *Xenopus* larvae.

## Materials and methods

### *Xenopus* embryos

Adult *Xenopus laevis* were purchased from a local farm (Hamamatsu Seibutsu Kyozai Co. Ltd; Shizuoka Prefecture, Japan), and fertilized eggs were obtained in the laboratory via artificial fertilization. Fertilized eggs were then placed in fresh water and incubated at 20 °C. Embryonic stages were determined based on Nieuwkoop and Faber (1967) [36]. The studies were performed according to the Ethical Guidelines for Animal Use of the Animal Care Committee at Ehime University.

### Isolation of axon-guidance genes in *X. laevis*

*Xenopus* homologues of *slit2* and *robo2* were isolated by polymerase chain reaction (PCR) using *X. laevis* embryonic cDNA as a template. The primers of *Xlslit2* were designed based on a published sequence (NM_001087668.1). The following primers were used: *Xlslit2*-F, 5’–TGAATCAGCACCACCAATGG–3’, *Xlslit2*-R, 5’–CTAGTCTCGATACCTTCTCG–3’; *Xlrobo2*-F, 5’–TGGATTGTAGAGTGCTGAGG–3’, *Xlrobo2*-R, 5’–CACGGAGCAATGCTACTTCC–3’.

The PCR products included in the agarose gel were purified using the Wizard SV Gel and PCR Clean-Up System (Promega), and the DNA fragment was cloned into pGEM-T Easy (pGEM-T Easy Vector Systems; Promega).

### Injection of morpholinos

To inhibit Slit2 or Robo2 signals specifically in neuronal tissues, a *slit2* MO (GCCACCCAAGGAAAGAACCCAACCA; Gene Tools, LLC) and a *robo2* MO (AGCCACCAGAAAGCCCATGTTTCCC) were injected at the 8-cell stage into the small blastomere of the animal pole, which differentiates into the CNS [37]. To visualize injected cells, enhanced green fluorescent protein (eGFP) mRNA was co-injected into the blastomere. Morpholinos containing five mismatched nucleotides were used as a control. As controls of *slit2* and *robo2*, GCgAgCCAAcGAAAcAAgCCAACCA and AGCCACgAcAAAcCCgATcTTTCCC were used, respectively (the mismatched nucleotide is shown in lower case). We injected 2.5 pmol of *slit2*, *robo2*, and control MOs. Injected embryos were incubated in 3 % ficoll until the blastula stage; subsequently, embryos were replaced in 0.3 × MMR (100 mM NaCl, 5 mM MgCl_2_, 0.5 mM CaCl_2_, 5 mM EGTA, 20 mM HEPES-NaOH, pH 7.5) at 20 °C. At stage 28, we checked eGFP illumination under a fluorescence microscope (Lumar V12; Carl Zeiss SMT GmbH, Oberkochen, Germany). Embryos showing neuron-specific localization of eGFP were incubated and used for further analyses.

### Morphological observation

Paraformaldehyde (PFA)-fixed specimens were observed under a stereomicroscope (Lumar V12; Carl Zeiss). The body length, curvature of the body axis, and surface area of the eyes were measured using the Axio Vision software (release 4.7.2; Carl Zeiss). Ten larvae were examined in each measurement.

### Behavioral analysis

**Fig. 1:**
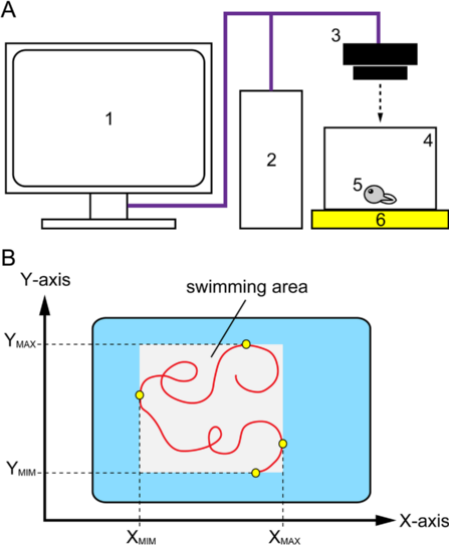
Experimental design of behavioral analysis and swimming trajectory. **a** Schematic diagram of the experimental apparatus: 1, monitor; 2, computer system; 3, camera; 4, glass aquarium; 5, biological sample (*Xenopus* larvae); 6, illuminator. **b** Quantification of swimming behavior. The maximum and the minimum values of the swimming trajectory (red line) in both the X- and Y-axis (X_MAX_, X_MIM_, Y_MAX_, Y_MIM_) were obtained from the recorded trajectory. Swimming area (light gray) is derived from max-min value of swimming trajectory

Behavioral analyses at the early larval stage (stage 46–47) were performed as described previously [38], with minor modifications (Fig. 1). A hatched larva was transferred to a small aquarium (130 × 150 mm) on a light board, and its swimming behavior was recorded for 3,000 frames (0.03 s/frame) using a video camera placed above the dish (Himawari GE60; Library Co. Ltd, Tokyo, Japan). Fifteen organisms were examined per experiment. The swimming trajectory was visualized on a personal computer, and the swimming speed and distance were measured using the Move-tr/2D7.0 software (Library Co. Ltd). To calculate the swimming pattern, the maximum and the minimum values of the swimming trajectory in both the x- and the y-axis were obtained as the swimming area (see also [38]). The whole experiment was performed twice. Significant differences in each variable among the subunit types were examined by Scheffé test after one-way analysis of variance (ANOVA) to characterize each subunit type.

### Axonal labeling of the posterior commissure

For retrograde labeling of axons using NeuroVue, *Xenopus* larvae were dissected in phosphate-buffered saline (PBS, pH 7.5). After fixation in 4 % PFA in PBS, a small chip of NeuroVue Red (Funakoshi 24835) was inserted into the primordium of the pretectum. Larvae were then incubated at 37 °C for one week in 2 % PFA in PBS. Labeled specimens were dissected, and the isolated brain was sectioned at 50 μm using a vibratome (EM PRO7; Dosaka EM Co. Ltd, Kyoto, Japan) after embedding in 5 % agar. Sections were then observed under a confocal microscope (LMS 510 META; Carl Zeiss).

### Whole-mount in situ hybridization

For whole-mount *in situ* hybridization, after fixation in 4 % PFA, embryos were dehydrated in a graded series of methanol (30 %, 50 %, 70 %, 90 %, and 100 %) and stored at −25 °C. *In situ* hybridization was performed as described previously [39], with minor modifications. After the color development, specimens were dissected, and the isolated brain was observed under a stereomicroscope (Carl Zeiss). Some specimens were cut into 50 μm using a vibratome after embedding in 5 % agar, and were then observed under a microscope (Axio Image A1; Carl Zeiss).

### Whole-mount immunohistochemistry

As a primary antibody to visualize developing axons, we used a monoclonal antibody raised against acetylated tubulin (T-6793, diluted 1/1000; Sigma-Aldrich). Developing muscles were stained with MF20, which recognizes myofilaments (obtained from the Developmental Studies Hybridoma Bank, University of Iowa; diluted 1/100). As a secondary antibody, we used Alexa Fluor 555 goat anti-mouse IgG (H + L; A21422; Invitrogen) or Alexa Fluor 488 goat anti-mouse IgG (H + L; A11001; Invitrogen). Nucleus was labeled with DAPI (D9564, 1 mg/mL, Sigma-Aldrich). For whole-mount immunostaining experiments, hatched larvae were prepared as described previously [38, 40]. The stained specimens were observed under a fluorescence microscope (Lumar V12; Carl Zeiss) or a confocal microscope (510 META; Carl Zeiss). Ten larvae were examined in each measurement. Statistical analyses were carried out using Student’s *t*-test.

### In situ hybridization combined with immunohistochemistry

Whole-mount *in situ* hybridization was performed as described above. Then, the samples were washed several times with Tris-buffered saline with 0.1 % triton-x100 (TBST). The nerve fibers were visualized by an immunohistochemistry by described above. Briefly, samples were incubated for 1 overnight in TBST containing 5 % skim milk (TSTM). They were then treated with the anti-acetylated tubulin antibody. Samples were incubated in the antibody for 2 days at RT in TSTM containing 0.02 % NaN_3_. The samples were then washed in TBST four times and subsequently incubated in the secondary antibody (Alexa488 anti-mouse IgG, diluted 1:500) for 2 days at RT in TSTM. The samples were then washed four times in TBST. The specimens were observed under a fluorescence stereomicroscope (Lumar V12; Carl Zeiss).

## Results

### Swimming behavior in MO-treated larvae

To study the molecular functions of *Xenopus* cognates of *slit2* (*Xlslit2*) and *robo2* (*Xlrobo2*) in the developing nervous system, MO were injected into the blastomere, which differentiates into the CNS. Although many MO-injected individuals showed no abnormal morphology at stage 44, a small number of larvae exhibited head curvature or eye reduction in both the control- and *slit2*-MO-injected groups. Conversely, the *robo2*-MO-injected specimens and their control-MO-injected specimens showed a more asymmetrical craniofacial morphology than did *slit2*-MO- and control-MO-injected specimens. Thus, in the subsequent analysis, we used *slit2*-MO-injected larvae and their control-MO-injected larvae (with normal body morphology), whereas we used *robo2*-MO-injected larvae and their control-MO-injected larvae, which have a slightly asymmetrical shape in the head or eye, in addition to larvae with normal body morphology.

**Fig. 2:**
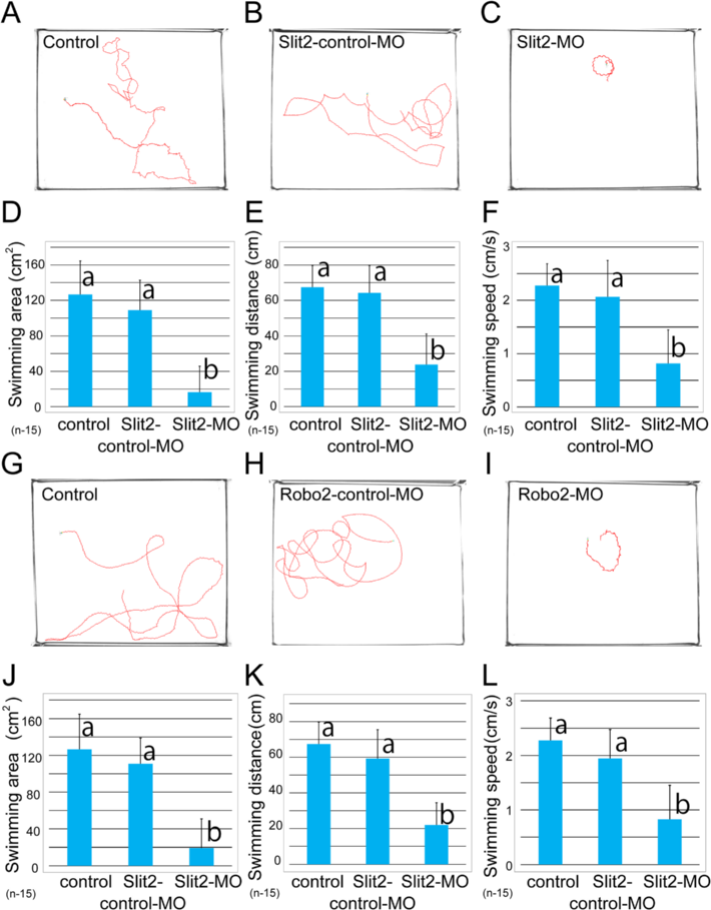
*slit2*- or *robo2*-MO-injected tadpoles indicates an abnormality in swimming behavior. **a–c, g–i** Red lines show the swimming trajectory of tadpoles: **a, g** Un-injected control, **b** Slit2-control-MO, **c** Slit2-MO, **h** Robo2-control-MO, **i** Robo2-MO. **d–f, j–l** Quantification of the swimming area (**d**, **j**), swimming distance (**e**, **k**) and swimming speed (**f, l**). In the *slit2*- or *robo2* MO-injected larvae, values of all items measured are significantly decreased. **d** The average swimming areas of control, Slit2-control-MO and Slit2-MO are 126.59 cm^2^, 108.92 cm^2^ and 16.40 cm^2^ respectively. **e** The average swimming distances of control, Slit2-miss-control and Slit2-MO are 67.44 cm, 64.24 cm and 23.83 cm respectively. **f** The average swimming speeds of control, Slit2-control-MO, and Slit2-MO are 2.28 cm/s, 2.06 cm/s and 0.82 cm/s, respectively. **j** The average swimming areas of control, Robo2-control-MO, and Robo2-MO are 126.59 cm^2^, 110.84 cm^2^ and 19.42 cm^2^, respectively. **k** The average swimming distances of control, Robo2-control-MO, and Robo2-MO are 67.44 cm, 59.24 cm and 21.99 cm, respectively. **l** The average swimming speeds of control, Robo2-control-MO, and Robo2-MO are 2.28 cm/s, 1.94 cm/s and 0.83 cm/s, respectively. Error bars are shown as standard deviation (SD). Data denoted by the same letter are not significantly different (P > 0.05) by Scheffé test after one-way analysis of variance

As *Slit2* or *Robo2* is involved in the formation of the early neuronal circuit in many vertebrates [27–30], we hypothesized that MO-treated larvae possess abnormalities not only in their neuronal elements, but in behavior as well. In fact, it has been reported that early-born neuronal frameworks play an essential role in the regulation of body movement [41]. Previous studies have shown that the first alternating body movements in *X. laevis* occur on both sides of the body during the early swimming stage (stages 28–33); however, the embryo does not move through the water. The myotomal musculature is fully developed during the free-swimming stage (from stage 33), and the embryo is able to move through the water and swim [33–35]. Larvae begin swimming to search for food at the early larval stage (from stage 45 on), at which point their oral apparatus becomes functional, enabling them to eat. As this free swimming is thought to be an initial behavior in this animal that is controlled mainly by early-born neuronal circuits, without modification by the postnatal experience, this swimming behavior is expected to be useful for the study of the function of neuronal networks constructed by an intrinsic genetic program. To test whether larvae treated with *Xlslit2* or *Xlrobo2* MO perform the correct movement, we analyzed the swimming pattern of larvae (stage 46–47) in which active swimming was continuously observed. Our behavioral analysis revealed that specimens that were treated with control MO swam in a large circular trajectory in a coordinated manner (Fig. 2b). In contrast, larvae treated with *Xlslit2* MO exhibited an abnormal swimming pattern of very small circles with unusual movements (Fig. 2c). A similar phenotype was observed in *Xlrobo2*-MO-treated larvae (Fig. 2i). The swimming areas traversed by larvae treated with *Xlslit2* and *Xlrobo2* MO were significantly smaller than those of control animals (Fig. 2d, j). In addition, the overall swimming distance and speed were also decreased significantly in *Xlslit2*- and *Xlrobo2*-MO-injected larvae compared with the controls (Fig. 2e, f, k, l).

### External morphology and musculature construction

**Fig. 3:**
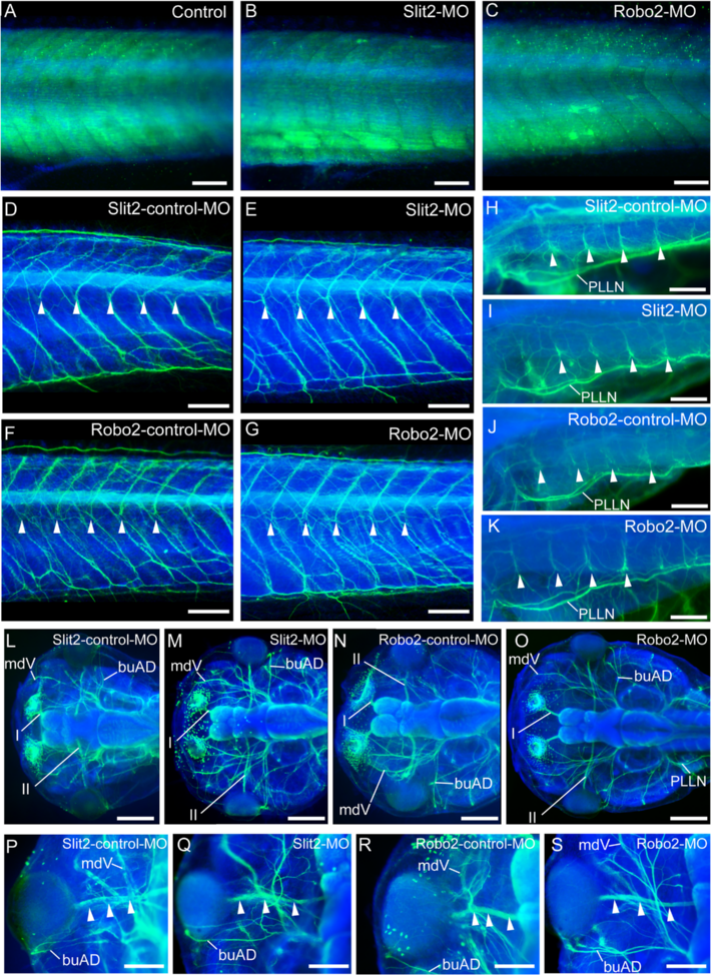
External morphology of *Xenopus* larvae. Blue staining show nucleus labeled by DAPI. Myotomes (**a–c**) and nerves (**d–s**) are visualized by immunohistochemistry (shown in green). **a–c** Lateral view of the trunk in un-injected control (**a**), *slit2*-MO-injected (**b**) and *robo2*-MO-injected (**c**) specimens. Development of myotomes is normal in all conditions. **d–g** Lateral view of the trunk in *slit2*-control-MO-injected (**d**), *slit2*-MO-injected (**e**), *robo2*-control-MO-injected (**f**), *robo2*-MO-injected (**g**) specimens. Arrowheads indicate segmentally organized spinal nerves. **h–k** Dorsal view of the anterior trunk in *slit2*-control-MO-injected (**h**), *slit2*-MO-injected (**i**), *robo2*-control-MO-injected (**j**), *robo2*-MO-injected (**k**) specimens. Arrowheads indicate segmentally organized spinal nerves. **l-o** Dorsal view of the head in *slit2*-control-MO-injected (**l**), *slit2*-MO-injected (**m**), *robo2*-control-MO-injected (**n**), *robo2*-MO-injected (**o**) specimens. **p-s** Dorsal view of the optic and cranial nerves in *slit2*-control-MO-injected (**p**), *slit2*-MO-injected (**q**), *robo2*-control-MO-injected (**r**), *robo2*-MO-injected (**s**) specimens. Arrowheads indicate the optic nerves. The peripheral nerves visualized by immunohistochemistry represent a normal innervation pattern. Scale bars: A–K, P-S, 200 μm; L-O, 500 μm

We next studied the morphology of the larvae used in the behavioral study to determine whether these larvae exhibited any morphological defects. Initially, we observed the trunk musculature and peripheral nerves, both of which are thought to be important for coordinated swimming. Immunostaining with an anti-myofilament antibody revealed that the segmented myomeres were arranged normally in the trunk region of *Xlslit2-* or *Xlrobo2-*MO-treated larvae, as well as in controls (Fig. 3a–c). In addition to the muscular system, *Xlslit2-* or *Xlrobo2-*MO-treated larvae exhibited a normal innervation pattern of the spinal nerves in the dorsal part of the trunk (arrowheads in Fig. 3d-g). High magnification images showed the segmentally innervating spinal nerves between myomeres (Fig. 3h-k). Furthermore, *Xlslit2-* or *Xlrobo2-*MO-treated larvae represented apparently normal morphology of the craniofacial peripheral nerves compared to those of control specimen (Fig. 3l–o), and we did not observe abnormal morphology of the cranial and the optic nerves in MO-treated larvae (Fig. 3p-s). Overall, there were no severe morphological problems in the musculature and peripheral nerves in the larvae used in the behavioral study, although, as noted above, some *Xlrobo2*- and its control-MO-injected specimens showed curvature of the brain and eye reduction (data not shown).

### Expression of Xlslit2 in *Xenopus* larvae

**Fig. 4:**
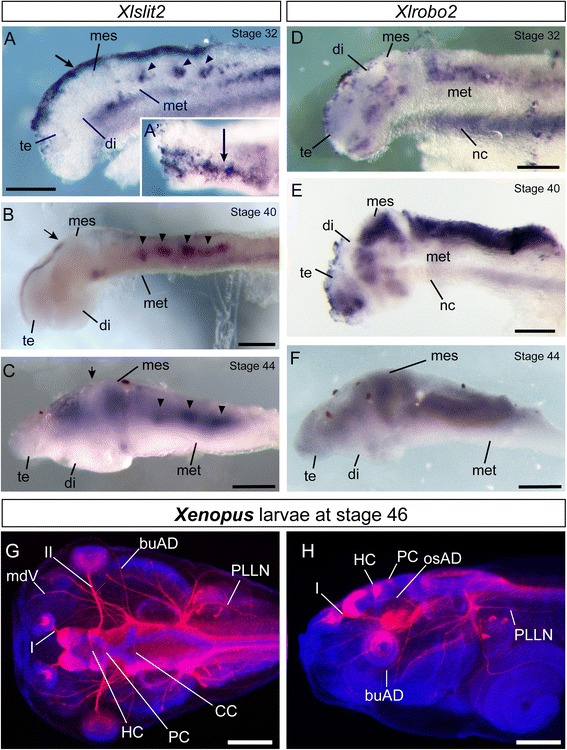
Expression patterns of *Xlslit2* and *Xlrobo2* and morphology of axonal tracts in *Xenopus* larvae. **a–c** Transcripts of *Xlslit2* are detected through the dorsal midline in the diencephalon at stage 32 (**a**), stage 40 (**b**) and stage 44 (**c**). *Xlslit2*-weak gap is found between diencephalon and mesencephalon (arrows in A and C, A’ is a dorsal view). In the metencephalon (met), expression domains of *Xlslit2* are observed along the antero-posterior axis (arrowheads). **d–f** Expression pattern of *Xlrobo2* at stage 32 (**d**), stage 40 (**e**) and stage 44 (**f**). (**d**) At early tail-bud stage, *Xlrobo2* is expressed in the telencephalon (te), diencephalon (di), mesencephalon (mes), dorsal metencephalon (met) and notochord (nc). **e, f** At middle and late tail-bud stage, *XlRobo2* expression is detected at high levels in the dorsal CNS. **g, h** Dorsal (**g**) and lateral (**h**) view of the developing *Xenopus* larva at stage 46. Axons in the PNS and CNS are visualized by anti-acetylated tubulin antibody. Habenular and posterior commissures are located the diencephalon (HC and PC). Scale bars: A-F, 200 μm; G and H, 500 μm

To identify the function of XlSlit2 and XlRobo2 on the process of formation of the neuronal circuit*,* we studied the expression patterns of transcripts encoding those genes in developing *Xenopus* embryos. At the early tail-bud stage (stage 32), *Xlslit2* was expressed in the alar plate throughout the telencephalon, diencephalon, and mesencephalon (Fig. 4a). However, *Xlslit2* expression domain in the dorsal diencephalon represented discontinuous pattern with many *Xlslit2*-weak gaps. One of these gaps (arrow in Fig. 4a’) appeared to be corresponded to the position of the posterior commissure (see below). *Xlslit2* was also located in the ventral region of the mesencephalon. In the metencephalon, *Xlslit2* expression domains manifest as a series of clusters located along the anteroposterior axis (arrowheads in Fig. 4a), one of which may correspond to the facial motor nucleus, as shown for *Slit2* expression in mouse [42]. At the middle tail-bud stage (stage 40), *Xlslit2* was expressed in the alar plate of the telencephalon and diencephalon, as in stage 32 (Fig. 4b). However, the expression level observed in the dorsal alar plate decreased at the dorsal midbrain; thus, the posterior limit of the expression domain was restricted at the posterior diencephalon (arrow in Fig. 4b).

In *Xenopus* embryos at late tail-bud stage (stage 44), *Xlslit2* transcripts were expressed at high levels in the dorsal part of the diencephalon, mesencephalon, and metencephalon (Fig. 4c). At this stage, *Xlslit2* expression regions became broader compared with those of stage 40 embryos, although the *Xlslit2* expression domain was still restricted to the specific part of the metencephalon (Fig. 4c). Conversely, *Xlslit2* transcripts were weakly expressed in the transitional region between the diencephalon and mesencephalon (arrow in Fig. 4c).

### Expression of Xlrobo2 in *Xenopus* larvae

Next, we observed the expression pattern of the *Xenopus* ortholog of *robo2* (*Xlrobo2*), which is a putative receptor of *Xlslit2.* At stage 32, *Xlrobo2* was expressed at high levels in the metencephalon (Fig. 4d). Furthermore, the *Xlrobo2* transcript was observed in the diencephalon and ventral telencephalon (Fig. 4d). At stage 40, the *Xlrobo2* mRNA was detected throughout the dorsal level of the brain (Fig. 4e)*.* In particular, it was expressed in the dorsal sides of the mesencephalon and metencephalon, whereas it was weakly expressed in the ventral neural tube. We did not detect *Xlrobo2* transcripts in the spinal cord (data not shown). At stage 44, *Xlrobo2* was expressed at higher levels throughout the dorsal part of the brain, as in the previous stage (Fig. 4f).

### Morphology of the nervous system in *Xenopus* larvae

Immunostaining using an anti-acetylated tubulin antibody was performed to investigate the developmental process of the *Xenopus* nervous system. In the embryonic stage 46, several cranial nerves, including the olfactory (the first cranial nerve, I), the optic (the second cranial nerve, II), the trigeminal (mdV), the anterior lateral line (buAD), and the posterior lateral line (PLLN) nerves, were observed (Fig. 4g, h), as in matured tadpole larvae. We also identified spinal nerves arranged segmentally in the trunk region (data not shown). These nerves showed clear segregation and projected to their correct targets. Next, we studied the axonal organization of the CNS, which receives input from the peripheral nerves. At stage 46, we observed several longitudinal or commissural axonal bundles, in which three distinct commissural tracts were observed in the dorsal side of the neural tube (Fig. 4g, h). Among those, the HC was located in the anterior part of the dorsal diencephalon. The PC was observed in the posterior part of the dorsal diencephalon (apparently corresponding to the pretectum). The commissures in the cerebellum (CC), which may include commissure cerebelli and commissure vestibulo-lateralis [43], were located on the cerebellum and across the midline on the dorsal side.

### Expression of Xlslit2 and Xlrobo2 in relation to the commissural tracts

**Fig. 5:**
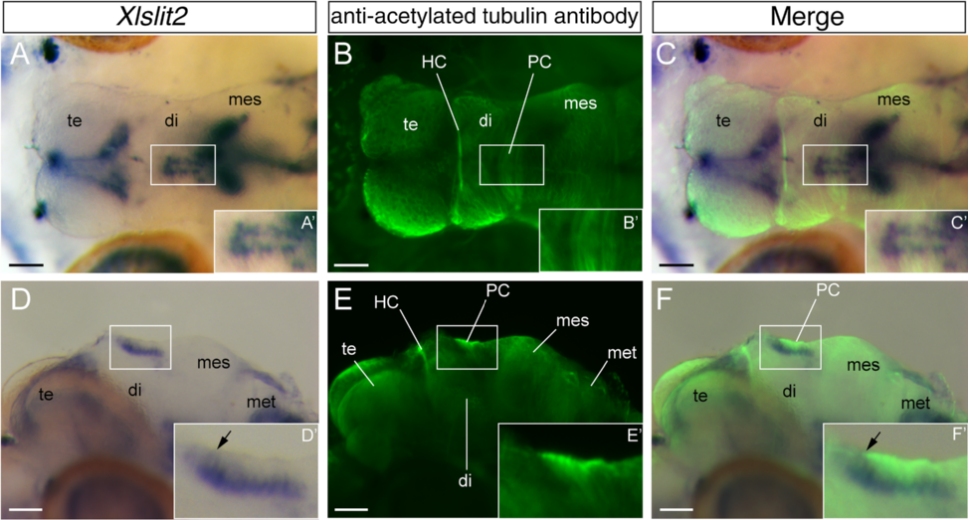
Morphology of axonal tracts and *Xlslit2* expression domain in the neural tube. Dorsal (**a-c**) and lateral (**d-f**) view of the developing *Xenopus* larva at stage 42. (A’-F’) High-magnification images of white squares in A-F. **a, c** Transcripts of *Xlslit2. Xlslit2* mRNA is discontinuously expressed in the dorsal diencephalon. The anterior limit of *Xlslit2* attach to the superficial region of the brain (arrow in D). **b, e** Axons in the CNS are visualized by immunohistochemistry (shown in green). Habenular and posterior commissures are visible. **c, f** Merged image of *Xlslit2* (purple) and nerves (green). The anterior end of the PC corresponds to the anterior limit of *Xlslit2* expression domain (arrow in F). Scale bars: 100 μm

**Fig. 6:**
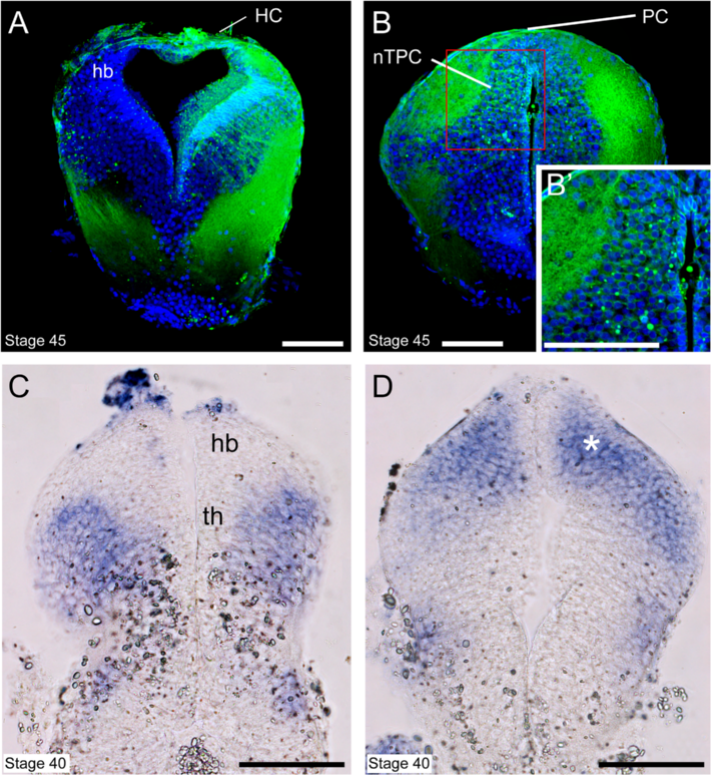
Morphology of axonal tracts and *Xlrobo2* expression domain in the diencephalon. **a, b** Coronal sections of the tadpole brain at stage 45, in which NeuroVue chips were inserted on the dorsal diencephalon and labeled neurons are shown in green. Blue staining shows nucleus labeled by DAPI. **a** Labeled axons (green), which correspond to the HC, are observed in the dorsal region of the diencephalon. **b** Labeled PC axons are observed in the dorsal diencephalon and labeled cell bodies are located ventral to the PC. This region is the presumptive nucleus of the tract of the PC (nTPC). (B’) High-magnification image of the red box in B. **c, d** Expression pattern of *Xlrobo2* on coronal sections of the tadpole brain at stage 40. **c** and **d** are the slice of anterior and posterior diencephalon, respectively. **d** *Xlrobo2* expression domain is observed in the nTPC (asterisk). Scale bars: 100 μm

We then compared the expression domains of *Xlslit2* and *Xlrobo2* in immunostained axon tracts, to determine whether *Xlslit2* and *Xlrobo2* expression domains correspond to the tracts of early-developing axons reported previously in *Xenopus* [1, 44, 45]. We initially studied the expression pattern of *Xlslit2* at stage 42. In this stage, *Xlslit2* transcripts represent a discontinuous expression pattern in the transitional region between the diencephalon and mesencephalon, where its expression domain is located in a slightly deeper region while its anterior limit reached to the superficial part (Fig. 5a, d, arrow in Fig. 5d’). Double labeling using DIG-labeled *Xlslit2* probe and anti-acetylated tubulin antibody revealed that the position of the *Xlslit2* expression domain was localized beneath the axon bundle of the PC, which crosses the midline on the dorsal diencephalon (Fig. 5b, c, e, f). It appears that PC axons path through *Xlslit2* weak region in the expression domain and its anterior end corresponds to the anterior limit of *Xlslit2* expression domain (Fig. 5a, c, c’, arrow in Fig. 5f’). In addition, the *Xlrobo2* mRNA was located in the ventral side of the PC, where axons contributing to the PC appear to originate. Thus, to clarify the anatomical position of *Xlrobo2*-expressing nuclei, we performed neuron labeling in the larval brain. As noted above, HC and PC were found on the anterior and posterior side of the dorsal diencephalon, respectively (Fig. 4g). Thus, NeuroVue was injected into the dorsal diencephalon, where HC and PC are thought to be located. We observed the retrogradely labeled axons of HC and PC in stage 45 larvae (Fig. 6a, b), and identified the labeled cell bodies in the section of the pretectum (posterior diencephalon). Comparison with adult brain morphology, the crop of cell bodies was thought to correspond to the nucleus of the tract of the posterior commissure (nTPC), which sends axons into the PC in many vertebrates [46] (Fig. 6b, b’).

Next, to identify the positional relationship between the expression domain of *Xlrobo2* and the nTPC, stained specimen were cut into coronal sections and were observed at stage 40. In the anterior diencephalon (presumptive thalamus), the expression domain of *Xlrobo2* was located in the ventral side, and it was not detected in the dorsal area, including the habenular nucleus (Fig. 6c). In contrast, in the section of the posterior diencephalon (pretectum), the *Xlrobo2* transcript was located in the dorsal part, corresponding to the nTPC (Fig. 6d, asterisk).

### Morphology of the developing CNS in MO-injected larvae

**Fig. 7:**
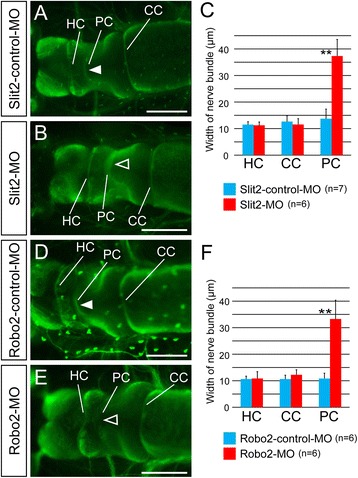
*slit2*/*robo2*-MO injected larvae change the width of the posterior commissure. **a, b, d, e** dorsal views of the tadpole brain at stage 44. **a** In *slit2*-control-MO injected larva, three commissures (habenular commissure: HC, posterior commissure: PC and cerebellar commissures: CC) are observed. PC indicated by an arrowhead. **b** In Slit2-MO injected larva, morphology of the habenular and cerebellar commissure appear to be normal, whereas nerve bundle of the posterior commissure become wider than the control larva (open arrowhead). **c** Quantification of the width of nerve bundle in Slit2-control- (blue)/Slit2-MO (red). *slit2*-MO-injected larva is significantly changed the width of the posterior commissure compared to that of *slit2*-control-MO (**P < 0.01). **d, e** In *robo2*-control- (D)/*robo2*-MO (**e**) injected larvae, the former represents three commissures but the latter shows unclear PC bundle (open arrowhead). **f** Quantification of the width of three commissures. The posterior commissure of *robo2*-MO-injected larva is significantly changed the width of the PC. Error bars are shown as standard deviation (SD). *P value was obtained by ANOVA (P < 0.05 is significant). (**P < 0.01). Scale bars: 200 μm

To identify morphological phenotypes of *Xlslit2*- and *Xlrobo2*-MO-injected larvae in the developing nervous system, we observed the overall morphology of the larval CNS in MO-treated specimens, and found a normal morphology for the telencephalon, diencephalon, mesencephalon, and metencephalon in both control-MO- and *Xlslit2*-MO- or *Xlrobo2*-MO-treated larvae at stage 44 (Fig. 7a, b, d, e). Next, to clarify whether some defects could be detected in the neuronal circuit, we observed the morphology of commissural tracts (HC, PC, and CC) on immunostained specimens. We found that the morphologies of the HC and CC in larvae treated with *slit2* or *robo2* MO were not significantly different from those of the control-MO-treated larvae, whereas the PC in *Xlslit2*-MO or *Xlrobo2*-MO-treated larvae exhibited an unclear commissural bundle on the dorsal diencephalon (Fig. 7b, e). This may be explained by the defasciculation of the tract, so that it could not be observed as a tight bundle. Thus, we then measured the width of bundles, and found that the PC in *Xlslit2*-MO-or *Xlrobo2*-MO-treated larvae was significantly wider than that of control larvae, whereas the width of the HC and CC was unaffected (Fig. 7c, f).

### Morphology of the PC in MO-treated embryos

**Fig. 8:**
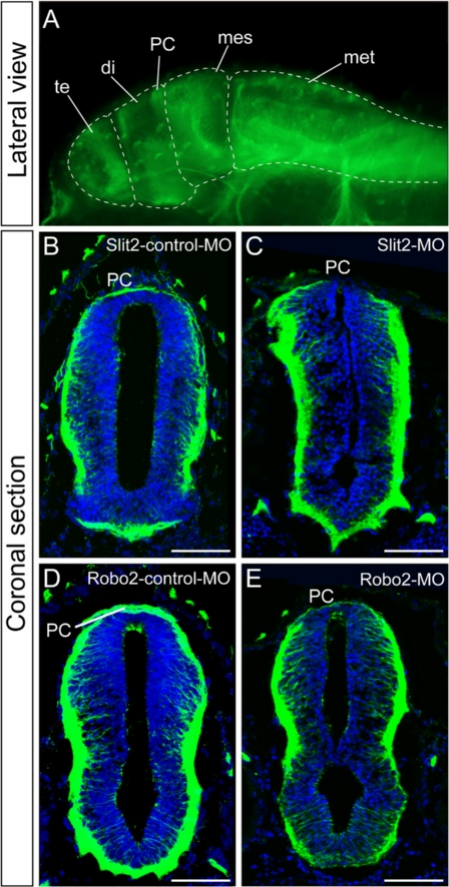
*slit2*/*robo2*-MO injected larvae represent the abnormal morphology of the posterior commissure. Neurons are visualized by immunohistochemistry (shown in green). **a** Lateral view of the brain in *Xenopus* larva. Dashed line indicates the outline of the brain regions. **b–e** Coronal sections at the level of the posterior commissure: **b** Slit2-control-MO, **c** Slit2-MO, **d** Robo2-control-MO and **e** Robo2-MO. In *slit2*/*robo2*-MO-injected larva, the bundle of the posterior commissure is thinner than that of control-MO. Blue staining show nucleus labeled by DAPI. Scale bars: 100 um

**Fig. 9:**
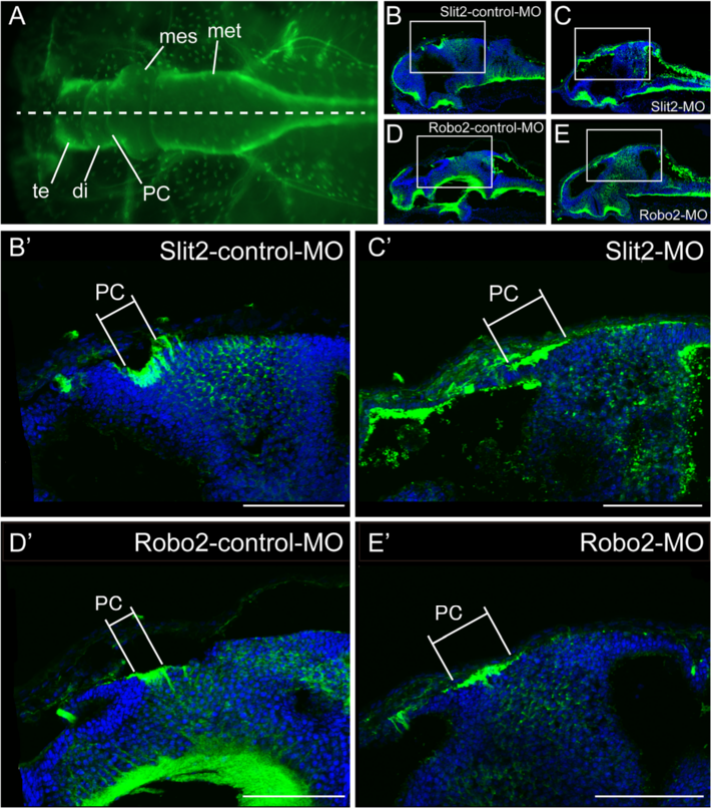
Median sections of the tadpole brain. Neurons are visualized by immunohistochemistry (shown in green). **a** Dorsal view of the *Xenopus* larva that performed the fluorescent immunostaining. Dashed line indicates the cutting plane in (**b–e**). **b’–e’** High magnification images of the box in (**b–e**): **b, b’** Slit2-control-MO, **c, c’** Slit2-MO, **d, d’** Robo2-control-MO, **e, e’** Robo2-MO. In the Slit2/Robo2-control-MO, the posterior commissures make a tight bundle, whereas in the Slit2/Robo2-MO injected larvae, the width of PC is elongated anteroposteriorly. Blue staining shows nucleus labeled by DAPI. Scale bars: 100 um

To clarify further the defect of the PC, we studied the morphology of the PC in coronal sections of embryos at stage 44 and found that the PC bundle in *Xlslit2*-MO- or *Xlrobo2*-MO-treated larvae became thinner on the dorsal diencephalon compared with those of control specimen (Fig. 8). Similarly, in the sagittal sections, the PC in *Xlslit2*-MO- or *Xlrobo2*-MO-treated larvae became thinner in dorso-ventral axis, and exhibited an anteroposteriorly elongated morphology (Fig. 9).

Overall, the results of this series of experiments suggest that Slit2 and/or Robo2 is specifically involved in the fasciculation of the PC.

## Discussion

In the present study, we focused on the function of Slit2 and Robo2*,* both of which play crucial roles in the patterning of CNS axons [47], and identified three novel findings. First, expression patterns of *Slit2* and *Robo2* are spatiotemporally regulated. Second, *slit2*-MO- and *robo2*-MO injected larvae exhibited abnormal swimming behavior. Third, *slit2*-MO- and *robo2*-MO-injected larvae showed an abnormal morphology of the PC, a part of the embryonic axonal tract. These results suggest that the Slit2–Robo2 interaction primarily or secondarily affects to the neuronal circuitry that triggers the coordinated swimming pattern.

### Involvement of *Slit2* and *Robo2* in swimming behavior

We found that larvae treated with *slit2* and *robo2* MO exhibited an abnormal swimming trajectory. In addition, the swimming distance and speed of MO-injected larvae were also reduced, although control-MO-injected larvae showed no apparent defects in swimming behavior. We also found that *slit2*-MO- and *robo2*-MO-treated larvae showed a normal body musculature. These data suggest that Slit2 and Robo2 inhibition induced the abnormal swimming pattern observed, without disturbing basic body apparatuses, such as the fins or body muscles. Importantly, as noted above, both *Xlrobo2*-MO- and control-MO-treated larvae included specimens that exhibited abnormal morphology of the head and eye. Despite the presence of these head abnormalities in both control-MO- and *robo2*-MO-treated larvae, we observed a significant abnormality in *Xlrobo2-*MO-treated specimens regarding swimming behavior, whereas no significant problem was detected in the control group. Given that MOs were specifically injected into blastomeres, which differentiate into the nervous system, we speculated that MO-injected larvae exhibited nervous system defects in circuits that control body movement.

### Peripheral nerves in MO-treated larvae

We found that larvae treated with *slit2* and *robo2* MO exhibited an apparent normal morphology in the peripheral nerves including cranial and spinal nerves. Although one previous report indicated that central projection of the optic nerve in zebrafish robo2 mutant was abnormal [48], we did not find any abnormality of the optic nerve bundle projecting to the brain. However, as we could not follow the trajectory of the optic nerve toward the optic tectum, further experiment by axon labeling will be needed to identify whether MO-treated larvae exhibit abnormalities in the optic tract.

### Expression of *slit2* and *robo2* in developing *Xenopus* larvae

To clarify whether MO-treated specimens have any problem in the neurodevelopmental process, we studied the expression pattern of the *slit2* and *robo2* transcripts in relation to the developmental position of the commissural tracts.

Previous studies have showed that the *Slit2* and *Robo2* mRNAs are expressed in the developing nervous system in many vertebrates [21–24, 27–30]. The present study showed that *Xlslit2* was expressed in a specific part of the developing CNS, as in other vertebrates [23, 24, 49, 50]. We found that the expression domains of *Xlslit2* and *Xlrobo2* corresponded to the region in which the PC is formed; the *Xlslit2* expression domain located beneath the PC, and *Xlrobo2* was expressed in the nTPC, which may be a source of the PC (see below). Importantly, *Xlslit2* domain showed a discontinuous pattern in which PC appears to be extended in *Xlslit2-*weak region, and the anterior limit of *Xlslit2* expression domain corresponds closely to the anterior border of the PC. In addition to the spatial distribution, the temporal timing of *Xlslit2/Xlrobo2* expression was though to correspond to the development of the PC. Namely, the early scaffold of *Xenopus laevis* is formed at stage 32 [1], a time at which the PC (called TPC in *Xenopus*) is across the midline of the dorsal diencephalon. Our study showed that the expression of *Xlslit2* and *Xlrobo2* was observed at stage 32, which corresponds to the onset of PC formation. *Xlslit2* and *Xlrobo2* were also expressed at stage 40, a time point at which the early tract is being constructed. Subsequently, the *Xlslit2* and *Xlrobo2* transcripts were observed at stage 44, a stage at which tight bundle of PC could be observed. Thus, *Xlslit2* and *Xlrobo2* were expressed correspondingly to the developmental time course of the PC.

### Interaction between *slit2* and *robo2* in the formation of the PC

The present study revealed that, although the HC and CC showed normal morphology, the PC exhibited an abnormal morphology in both *slit2*-MO- and *robo2*-MO-injected larvae. This may be due to a problem in the fasciculation process; the PC bundle in MO-treated specimens was thinner and wider compared with that of control larvae. Previous studies using insects and vertebrates showed that Slit2 and Robo2 are involved in the formation of axon bundles [22, 23] and commissural tracts [25–30, 32]. Those findings support our present finding that *Xenopus* cognates of Slit2 and Robo2 seem to be involved in the formation of the PC.

**Fig. 10:**
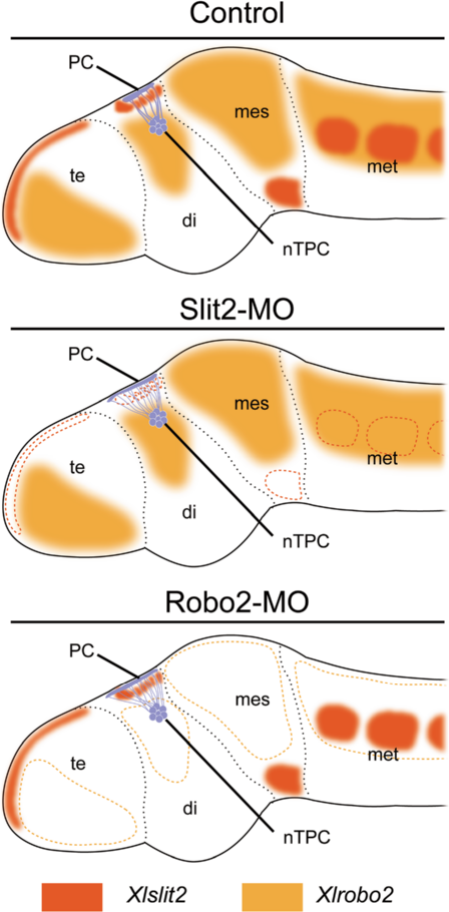
Schematic view of the result of the experiment. In the control, Robo2-expressing PC axons extend through the Slit2-weak region in the posterior diencephalon and result in a tight bundle. However, in the *slit2-*/*robo2*-MO-injected larvae, the PC makes elongates anteriorly with a defasciculated tract, due to the absence of repulsive interaction between Slit2 and Robo2. Disappearance of gene expression indicated by the dashed line

Regarding the molecular mechanism underlying PC formation in *Xenopus*, the present study showed that the *Xlrobo2* expression domain in the diencephalon appeared to correspond to the region that includes the nTPC. Because *slit2*-MO- and *robo2*-MO-injected larvae showed a very similar phenotype, XlSlit2 may act as a ligand for XlRobo2 during the formation process of the PC. Thus, it is speculated that XlRobo2-expressing PC axons may avoid the XlSlit2 protein secreted from the pretectum, thus allowing axons to pass through the Slit2-negative or Slit2-weak region. We found that *Xlslit2* mRNA exhibits discontinuous expression beneath the PC, and PC axons appear to run through an *Xlslit2*-weak region. This morphological relationship between *Slit2* and PC axons has not been reported in other vertebrates. This finding may thus provide new insights into the function and evolution of Slit-Robo signaling. Importantly, anterior domain of *Xlslit2* extended dorsally and attached to the superficial region of the brain where the anterior border of the PC was formed. In the *slit2*-MO-injected larvae, the anterior border of PC seemed to be extended toward the anterior neural tube, which suggests that the PC axons are repelled by XlSlit2 in the superficial region of the diencephalon, resulting in a tight bundle on the posterior side of the pretectum (Fig. 10); however, the manner in which the posterior limit of the tract is formed remains unknown. Importantly, despite the abnormalities in the PC in MO-treated embryos, we found a normal morphology for the other commissures (HC and CC) in *Xlslit2*-MO- and *Xlrobo2*-MO-treated embryos. This may be attributable to the absence of *Xlrobo2* transcripts in the nuclei that provide axons to those tracts. Other guidance molecule(s) may be involved in the formation of those commissures.

### Evolutionary perspective of Slit-Robo signaling in the formation of the PC

**Fig. 11:**
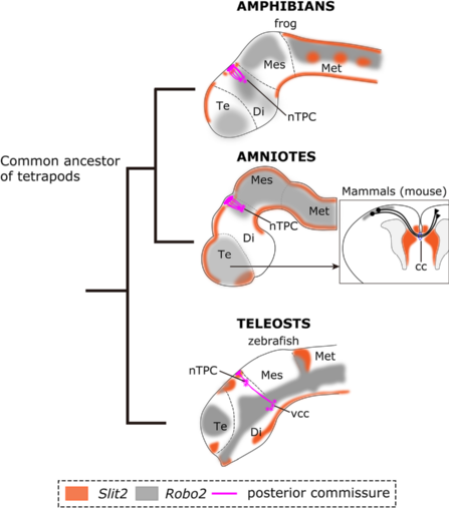
Evolutionary process of Slit/Robo mediated axonal wiring. In amphibians and amniotes, Slit2-weak region is present in the superficial part of the diencephalon, in which posterior commissure is formed. Robo2 is expressed in axons of the posterior commissure originates from nTPC in the posterior diencephalon. In the zebrafish (24 h post fertilization), slit2 expression domain in the dorsal diencephalon is similar to those of tetrapods, whereas robo2 transcripts are appear to be absent in the homologous region of nTPC. In mammals, Slit/Robo is also used for the development of the newly acquired commissure, such as corpus callous (indicated by the blue dashed ring). cc, corpus callous; Di, diencephalon; Mes, mesencephalon; Met, metencephalon; nTPC, nucleus of the tract of the posterior commissure; PC, posterior commissure; Te, telencephalon; vcc, ventrocaldal cluster

Recent studies have shown that in amniotes (chick and mouse), PC located to slit2 weak region on the dorsal diencephalon neighboring to the anterior midbrain [23, 49]. In addition, Robo2 transcripts appear to be expressed in the region corresponding to the nTPC in mice [23, 51, 52]. These morphological characters are markedly similar to those in *Xenopus laevis* represented in our present study. On the other hand, although the expression domain of Slit2 cognate in zebrafish appeared to correspond to those of other amniotes [24, 53], expression pattern of zebrafish Robo2 seems to be different from those of amniotes [54]. Namely, its transcripts appeared to be absent in the nTPC [22]. Taken together, origin of Slit –Robo signaling that contributes to the PC formation may date back to the common ancestor of tetrapods (Fig. 11). However, abnormality in the PC has not been reported in other amniotes including slit or robo mutant mice [27, 28, 30]. It may be due to the fact that the disruption of slit or robo signaling may be compensated for by other paralogues or guidance molecules, such as the EphA7-dependent system which is involved in the formation of the chick PC [55]. Thus, further functional studies using vertebrate groups should be necessary to identify the evolutionary process of PC formation. Consequently, Slit2-Robo2 signaling might contribute to the generation of the novel commissural system which participated in the evolution of higher cognitive center, as Slit2-Robo2 signaling is involved in the formation of corpus callosum (cc; [28]), a well developed commissural system, which thought to be newly established in eutherian mammals. Since the cc plays a key role in the cognitive function in the mammalian neocortex, establishment of cc may be an important step in the evolution of the neocortex as the highest integrative center.

### Neuronal defects may contribute to abnormal behavior

It is reasonable to speculate that, if *Xlslit2*-MO- and *Xlrobo2*-MO-treated larvae exhibit defects in the neuronal circuitry of the CNS, they would show some kind of defect in motor movement, due to the disruption of neuronal wiring. In fact, we found that *Xlslit2*-MO and *Xlrobo2*-MO-injected larvae showed an abnormal swimming pattern. As this behavior is thought to be an initial form of free swimming in *Xenopus* larvae, it may be controlled mainly by a neuronal circuit constructed by an intrinsic neurodevelopmental program. Thus, the Slit–Robo interaction appears to be involved in the establishment of functional brain element(s) that are formed in the embryonic or early larval stage and regulate an initial free swimming. Because these molecules are expressed at early stages, this behavioral defect may originate from the disruption of an early developing neuronal circuit that induces the coordinated swimming pattern. Otherwise, the later-developing axons that follow early-developing tracts constructed by Slit2–Robo2 signaling may contribute to the behavioral problem detected. Previous studies showed that inhibition of Slit2 or Robo2 results in an abnormal morphology of the TPOC in zebrafish and mouse [21–23]. Thus, *Xlslit2*-MO- and *Xlrobo2*-MO-injected *Xenopus* embryos may develop abnormalities in the TPOC itself, as in other vertebrates, which may cause behavioral abnormalities. Otherwise, the neuronal circuits that are formed subsequently on the framework of the TPOC may be related to the behavioral problem observed in the present study, although we were unable to check abnormality in the TPOC using these morphants due to technical difficulties (unavailability of specific antibodies that recognize TPOC). In addition, the commissural interneurons in the spinal cord that produce appropriate muscle contraction and body movements may be affected by *Xlslit2*-MO- and *Xlrobo2* inhibition, although transcripts of *Xlslit2* and *Xlrobo2* could not be observed in trunk myomeres and the spinal cord, respectively. However, as observed in the present study, abnormal morphology of the PC may induce behavioral changes. Although it is unknown whether there is a direct link between the PC and swimming behavior, it is important to note that axons in the PC stem from several types of neurons. Previous studies have shown that the PC contains axons that stem from several nuclei, including the nTPC [46, 55]. In mammals, the PC is involved in oculomotor movement by transmitting visual information coming from the cerebral cortex (visual area) and superior colliculus [56, 57]. Moreover, in mammals and birds, the PC contains neurons from the medial longitudinal fascicle (MLF), which includes a neuronal circuit involving the coordination of sensory and motor nerve integration. In chicks, neurons consisting of the PC (TPC) were located within the MLF, intermingled with the central and dorsal populations of MLF neurons [58]. These data suggest that the guidance mechanism for visual and/or MLF axons is affected by MO treatment and, hence, fails to form functional neuronal connections. Therefore, we surmise that the malformed sensory circuits in the PC would be a suitable as a candidate that caused the abnormal behavior in the *slit2*-MO- and *robo2*-MO-treated larvae. Conversely, it is possible that the other neuronal system that is formed by the interaction between Slit2 and Robo2 results in the behavioral problem. Future neuroanatomical and functional studies are necessary to identify the molecular mechanism via which correct neural circuits and behavioral patterns are elicited.

## Abbreviations

buAD: buccal ramus of anterodorsal lateral line nerve
CC: Commissures in the cerebellum
di: diencephalon
hb: habenular
HC: Habenular commissure
mes: mesencephalon
met: metencephalon
mdV: mandibular division of the trigeminal nerve
mxV: maxillary division of the trigeminal nerve
nc: notochord
osAD: superficial ophthalmic ramus of anterodorsal lateral line nerve
PC: Posterior commissure
PLLN: Posterior lateral line nerve
te: telencephalon
th: thalamus
Sn: spinl nerve
I: olfactory nerve
II: optic nerve. Schlosser and Northcutt (2000) was referred for the morphological identification [59]
